# Association of Gas Diffusing Capacity of the Lung for Carbon Monoxide with Cardiovascular Morbidity and Survival in a Disadvantaged Clinical Population

**DOI:** 10.1007/s00408-022-00580-9

**Published:** 2022-10-22

**Authors:** Andrew J. Collaro, Anne B. Chang, Julie M. Marchant, Mark D. Chatfield, Annette Dent, Kwun M. Fong, Margaret S. McElrea

**Affiliations:** 1 Department of Respiratory and Sleep Medicine, Queensland Children’s Hospital, Level 5a, 501 Stanley St, South Brisbane, QLD 4101 Australia; 2 Australian Centre for Health Services Innovation, Queensland University of Technology, Brisbane, QLD Australia; 3 Child Health Division, Menzies School of Health Research, Casuarina, NT Australia; 4 Indigenous Respiratory Outreach Care, The Prince Charles Hospital, Brisbane, QLD Australia; 5 Thoracic Medicine, The Prince Charles Hospital, Brisbane, QLD Australia; 6 Faculty of Medicine, The University of Queensland, Brisbane, QLD Australia; 7 UQ Thoracic Research Centre, Faculty of Medicine, The University of Queensland, Brisbane, QLD Australia

**Keywords:** *D*_LCO_, *K*_CO_, *V*_A_, Gas diffusing capacity, Mortality, Cardiovascular

## Abstract

**Purpose:**

Low diffusing capacity of the lung for carbon monoxide (*D*_LCO_) and spirometry values are associated with increased mortality risk. However, associations between mortality risk and cardiovascular disease with the transfer coefficient of the lung for carbon monoxide (*K*_CO_) and alveolar volume (*V*_A_) are unknown. This cohort study: (i) evaluated whether *D*_LCO_, *K*_CO_, and *V*_A_ abnormalities are independently associated with cardiovascular morbidity and/or elevated mortality risk and, (ii) compared these associations with those using spirometry values.

**Methods:**

Gas-diffusing capacity and spirometry data of 1165 adults seen at specialist respiratory outreach clinics over an 8-year period (241 with cardiovascular disease; 108 deceased) were analysed using multivariable Cox and logistic regression.

**Results:**

*D*_LCO_, *K*_CO_, and *V*_A_ values below the lower limit of normal (< − 1.64 *Z*-scores) were associated with elevated cardiovascular disease prevalence [respective odds ratios of 1.83 (95% CI 1.31–2.55), 1.56 (95% CI 1.08–2.25), 2.20 (95% CI 1.60–3.01)] and increased all-cause mortality risk [respective hazard ratios of 2.99 (95% CI 1.83–4.90), 2.14 (95% CI 1.38–3.32), 2.75 (95% CI 1.18–2.58)], after adjustment for factors including age, personal smoking, and respiratory disease. Compared to similar levels of spirometry abnormality, *D*_LCO_, *K*_CO_*,* and *V*_A_ were associated with similar or greater mortality risk, and similar cardiovascular disease prevalence. Analysis of only those patients with clinical normal spirometry values (*n* = 544) showed these associations persisted for *D*_LCO_.

**Conclusion:**

Low *D*_LCO_, *K*_CO_, and *V*_A_ measurements are associated with cardiovascular disease prevalence. As risk factors of all-cause mortality, they are more sensitive than spirometry even among patients with no diagnosed respiratory disease.

## Introduction

There is a growing evidence that low spirometric lung function is independently associated with increased future cardiovascular morbidity [1, 2], and overall mortality [1–4]. These associations have been demonstrated in asymptomatic patients without respiratory disease [4], and in patients whose spirometry *Z*-scores are reduced below zero but still within the clinically normal range (0 to − 1.64 *Z*-scores) [2, 5]. Comparatively fewer studies have reported on the association between diffusing capacity of the lung for carbon monoxide (*D*_LCO_) and mortality [6, 7]. Limited existing data suggests clinically abnormal *D*_LCO_ is associated with elevated mortality risk with greater effect size than for spirometry [6, 7], even in the absence of diagnosed respiratory disease [6]. As yet, no studies have reported on associations between *D*_LCO_ and overall cardiovascular morbidity. Furthermore, other key parameters of the diffusing capacity test, i.e., the transfer coefficient of the lung for carbon monoxide (*K*_CO_) and alveolar volume (*V*_A_), remain entirely unexplored for their association with either overall cardiovascular morbidity or all-cause mortality.

*K*_CO_ is the rate of carbon monoxide (CO) removal from alveolar gas during the single breath hold manoeuvre [8], and is thus the change in log concentration of CO in the inspired gas volume (*V*_I_) divided by the breath hold time (10 ± 2 s [9] for an acceptable manoeuvre). Despite controversy surrounding the diagnostic utility of the *K*_CO_ [10–12], it is useful in discerning patterns of parenchymal or obstructive pathologies [11, 13]. *V*_A_ is an estimate of the volume of gas accessible to surfaces of gas exchange (the respiratory membrane that forms the alveolar wall) and equates to *V*_I_ minus the volume of gas remaining in the large and small airways that does not participate in gas exchange [8]. The product of the *K*_CO_ and *V*_A_ is then adjusted for pressure to give the *D*_LCO_. The *K*_CO_ and *V*_A_ provide complementary information to the *D*_LCO_ and afford insight into the root cause of *D*_LCO_ abnormality, and both should be reported alongside the *D*_LCO_ according to the current standard [9]. Given the clinical utility of these measurements and their availability to clinicians interpreting test results, assessing whether these parameters are also associated with future mortality risk would add valuable knowledge.

We utilised the data from regional and remote clinical populations seen and treated at specialist respiratory outreach clinics over an 8-year period to evaluate whether *D*_LCO_, *K*_CO_, and *V*_A_ abnormalities are associated with future outcomes. The majority of participants were First Nations Australians who are, compared to non-First Nations people, at significantly increased risk of chronic respiratory [14] and cardiovascular disease [15] amongst other health disparities [16]. Investigating this question in an at-risk population is important, as such investigations are more likely to uncover significant associations. The objectives of our study were to (i) determine whether *D*_LCO_, *K*_CO_, and *V*_A_ abnormalities are associated with cardiovascular morbidity and/or elevated mortality risk independent of other factors e.g., concurrent respiratory disease, and (ii) compare these associations with those involving FEV_1_, FVC, and FEV_1_/FVC values obtained from spirometry testing.

## Materials and Methods

### *Study Design*,* Setting*,* and Participants*

This is an 8-year retrospective analysis of adults referred to Indigenous Respiratory Outreach Care (IROC) clinics in regional and remote Queensland through their primary care physicians, community health workers, or self-referral. Inclusion criteria were adults aged 18–85 years at IROC clinic visit, who were medically reviewed with acceptable and repeatable [9] gas diffusing capacity and spirometry testing performed by a trained respiratory scientist between February 2012 and March 2020. Ethical approval for this study was granted by The Prince Charles Hospital Human Research Ethics Committee (Reference: HREC/2019/QPCH/58452).

### Lung Function Testing

Gas diffusing capacity (single breath) and spirometry testing were performed according to European Respiratory Society (ERS) and American Thoracic Society (ATS) criteria [9, 17]. Patients were tested using an EasyOne Pro Lab (ndd Medizintechnik). Global Lung Function Initiative (GLI) multi-ethnic reference equations for spirometry (GLI-2012) [18] were used to generate predicted values for spirometry tests using the ‘other/mixed’ category, as this was found to be most suitable for use in First Nations Australian children and young adults [19]. GLI reference equations for gas diffusing capacity testing (GLI-2017), including the 2020 corrections [20], were applied to tests to derive *Z*-scores for each patient’s values [18, 20]. Notably, no ethnic correction is available. We used the ATS/ERS [21] four-tier grading with approximated* Z*-score equivalents proposed by Miller and Cooper [7] where GLI-derived [20] *Z*-scores ≥ − 1.64 were graded as normal, − 1.64 to − 3 as mild, − 3 to − 5 as moderate, and < − 5 as severe [7].

### Data Verification and Definitions

Patient demographics, gas diffusing capacity and spirometry test data, cardiovascular disease, personal smoking status, household smoking data, and comorbidities recorded by a respiratory physician at baseline (first visit to IROC clinic) for each patient were collected. Mortality data was censored in August 2020, with both cause (where available) and date of death collected. Electronic and paper chart medical records were used to verify patient demographic, medical, personal smoking, and household smoking data.

Mortality data were collected from electronic medical records, using death certificates where available, or discharge summaries. Where cause of death was unclear or no information was available, cause of death was categorised as ‘unknown’. Chronic respiratory disease included respiratory physician diagnoses of chronic obstructive pulmonary disease, asthma, bronchiectasis, or other chronic pulmonary disease, and cardiovascular disease included ischaemic heart disease, congestive heart failure, stroke or transient ischaemic attack, and cardiac valve disease.

### Statistical Analysis

Gas-diffusing capacity and spirometry data at first visit to IROC was used as the baseline for each patient included in this analysis. Odds ratios (ORs) for cardiovascular disease were calculated for gas diffusing capacity and spirometry *Z*-score tiers using logistic regression. Hazard ratios (HRs) for mortality events were calculated for baseline gas diffusing capacity and spirometry status using a Cox proportional hazards (PH) model after testing the PH assumption through visual inspection of log–log plots. Adjusted survival curves were generated assuming a Weibull distribution. Variables with *p* < 0.2 for any level on univariable modelling were included in final adjusted models. These variables included age, sex, personal smoking status, body mass index (BMI), First Nations status, and respiratory disease. Global Wald testing was performed on categorical variables. Stata17 (StataCorp LLC) was used for statistical analyses; two-tailed *p* values < 0.05 were considered significant.

## Results

**Fig. 1:**
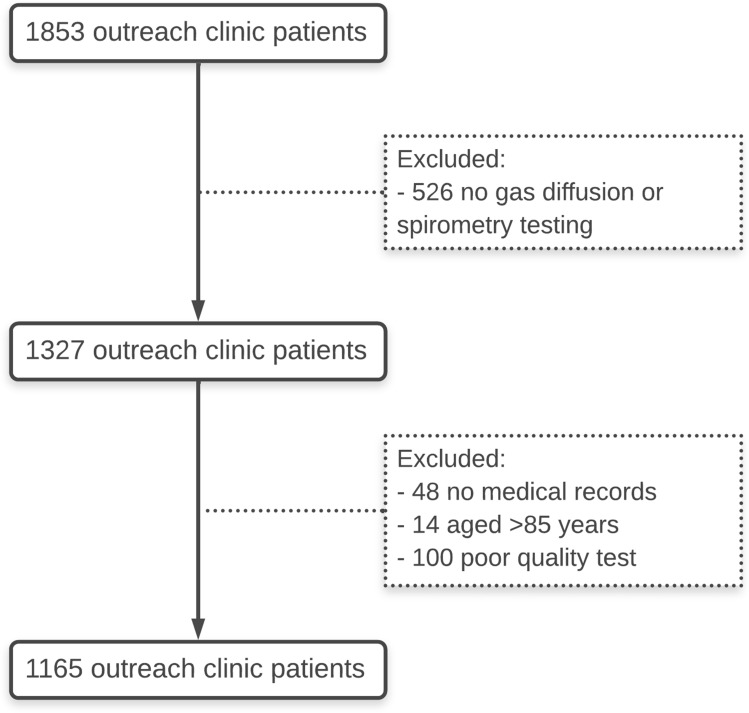
Summary of included outreach clinic patients

Over an 8-year period, 1853 patients were seen at adult IROC clinics. Of these, 1327 patients had gas diffusion capacity testing. We excluded 48 patients with no paper chart or electronic medical records, 14 patients aged > 85 years, and a further 100 who did not meet acceptability and repeatability criteria for gas diffusion capacity [9] or spirometry testing [17] (Fig. 1). Thus, a total of 1165 patients were included in this analysis where their demographic and gas diffusing capacity data are summarised in Table 1. The median time from baseline visit to data census was 3.5 years (IQR 2–5). Excluded participants were similar in age (59 years) with similar proportions of females (51%), were less likely to be current (24%) or former (24%) smokers but had similar cardiovascular disease prevalence (20%).

**Table 1:** Summary data of 1165 patients at initial specialist respiratory outreach clinic visit

	All patients (*n* = 1165)	Deceased patients (*n* = 108)
	Median (IQR) or *n* (%)	
Age, years	57 (48, 66)	66 (57, 75)
Female	651 (56%)	42 (39%)
First nations	763 (65%)	62 (57%)
Smoking status		
Current	434 (37%)	42 (39%)
Former	393 (34%)	39 (36%)
Never	248 (21%)	16 (15%)
Unknown	90 (8%)	11 (10%)
Body mass index (kg/m^2^)		
Underweight (< 18.5)	56 (5%)	12 (11%)
Healthy (18.5–24.9)	192 (16%)	30 (28%)
Overweight (25–29.9)	284 (24%)	35 (32%)
Obese (≥ 30)	633 (54%)	31 (29%)
Cardiovascular disease	241 (21%)	58 (54%)
Respiratory disease	904 (76%)	93 (86%)
*D*_LCO_		
*Z*	− 1.21 (− 2.37, − 0.11)	− 3.16 (− 4.77, − 1.79)
% Predicted	82 (67, 98)	57 (37, 73)
*K*_CO_		
*Z*	− 0.26 (− 1.51, 0.72)	− 2.07 (− 3.57, − 0.51)
% Predicted	96 (78, 111)	69 (49, 92)
*V*_A_		
*Z*	− 1.18 (− 2.03, − 0.31)	− 1.68 (− 2.70, − 0.91)
% Predicted	86 (77, 96)	81 (69, 89)
FEV_1_		
*Z*	− 1.62 (− 2.67, − 0.81)	− 2.64 (− 3.45, − 1.57)
% Predicted	76 (60, 89)	58 (41, 74)
FVC		
*Z*	− 1.27 (− 2.16, − 0.46)	2.10 (− 2.99, − 1.17)
% Predicted	82 (70, 94)	69 (56, 81)
FEV_1_/FVC		
*Z*	− 0.86 (− 2.05, − 0.08)	− 1.43 (− 3.28, − 0.08)
Raw	75 (65, 81)	67 (54, 79)

*D*_LCO_, diffusing capacity of the lung for carbon monoxide; *K*_CO_, transfer coefficient of the lung for carbon monoxide; *V*_A_, alveolar volume; FEV_1_ forced expiratory volume in one second; FVC forced vital capacity

Gas diffusing capacity values for included patients were below the lower limit of normal (− 1.64 *Z*-score) for 517 (40%) *D*_LCO_, 298 (23%) *K*_CO_, and 451 (35%) *V*_A_ results. Summary cardiovascular disease and mortality data for included patients are shown in Table 2.

**Table 2:** Summary baseline lung function grading data, cardiovascular disease, and subsequent survival data for 1165 patients seen at specialist respiratory outreach clinics

	Normal (*Z* ≥ − 1.64)	Abnormal (*Z* < − 1.64)		
		Mild (*Z* − 1.64 to − 3)	Moderate (*Z* − 3 to − 5)	Severe (*Z* < − 5)
*D*_LCO_				
* n* (%)	697 (60)	270 (23)	147 (13)	51 (4)
CVD (%)	103/697 (15)	57/270 (21)	64/147 (44)	17/51 (33)
Deaths (%)	24/697 (3)	24/270 (9)	37/147 (25)	23/51 (45)
*K*_CO_				
*n* (%)	901 (77)	149 (13)	97 (8)	18 (2)
CVD (%)	157/901 (17)	41/149 (28)	39/97 (40)	4/18 (22)
Deaths (%)	49/901 (5)	19/149 (13)	29/97 (30)	11/18 (61)
*V*_A_				
*n* (%)	753 (65)	324 (28)	84 (7)	4 (< 1)
CVD (%)	121/753 (23)	90/324 (28)	29/84 (35)	1/4 (25)
Deaths (%)	53/753 (7)	37/324 (11)	17/84 (20)	1/4 (25)
FEV_1_				
*n* (%)	590 (51)	364 (31)	200 (17)	11 (1)
CVD (%)	96/590 (16)	87/364 (24)	55/300 (28)	3/11 (27)
Deaths (%)	30/590 (5)	33/364 (9)	43/200 (22)	2/11 (18)
FVC				
*n* (%)	708 (61)	341 (29)	111 (10)	5 (< 1)
CVD (%)	115/708 (16)	90/341 (26)	35/111 (32)	1/5 (20)
Deaths (%)	40/708 (6)	41/341 (12)	26/111 (23)	1/5 (20)
FEV_1_/FVC				
*n* (%)	802 (69)	199 (17)	151 (13)	13 (1)
CVD (%)	158/802 (20)	42/199 (21)	40/151 (26)	1/13 (8)
Deaths (%)	60/802 (7)	19/199 (10)	26/151 (17)	3/13 (23)

CVD, cardiovascular disease; *D*_LCO_, diffusing capacity of the lung for carbon monoxide; *K*_CO_, transfer coefficient of the lung for carbon monoxide; *V*_A_, alveolar volume

### Cardiovascular Morbidity

A total of 241 (21%) of patients had diagnosed cardiovascular disease at baseline. Our adjusted models showed abnormal *D*_LCO_ (OR = 1.83, 95% CI 1.31, 2.55), *K*_CO_ (OR = 1.56, 95% CI 1.08, 2.25), and *V*_A_ (OR = 2.20, 95% CI 1.60, 3.01) were each associated with cardiovascular disease, independent of smoking status. Complete per-tier modelling is shown in Table 3.

**Table 3:** Unadjusted and adjusted ORs with 95% confidence intervals for cardiovascular disease from logistic regression and global Wald testing using *Z*-scores derived from GLI reference values [20]

		Unadjusted		Adjusted^a^	
	*n*	OR (95% CI)	*p*	OR (95% CI)	*p*
*D*_LCO_ (*Z*)					
Normal (≥ − 1.64)	697	Reference	< 0.001	Reference	< 0.001
Mild (− 1.64 to − 3)	270	1.54 (1.08, 2.21)		1.27 (0.86, 1.88)	
Moderate (− 3 to − 5)	147	4.45 (3.02, 6.55)		3.31 (2.12, 5.19)	
Severe (< − 5)	51	2.88 (1.55, 5.35)		2.77 (1.30, 5.91)	
*K*_CO_ (*Z*)					
Normal (≥ − 1.64)	901	Reference	< 0.001	Reference	0.03
Mild (< − 1.64 to ≥ − 3)	149	1.80 (1.21, 2.68)		1.28 (0.82, 2.00)	
Moderate (< − 3 to ≥ − 5)	97	3.19 (2.05, 4.95)		2.18 (1.30, 3.66)	
Severe (< − 5)	18	1.35 (0.44, 4.17)		1.41 (0.39, 5.09)	
*V*_A_ (*Z*)					
Normal (≥ − 1.64)	753	Reference	< 0.001	Reference	< 0.001
Mild (< − 1.64 to ≥ − 3)	324	2.01 (1.47, 2.74)		2.05 (1.46, 2.88)	
Moderate (< − 3 to ≥ − 5)	84	2.75 (1.69, 4.50)		2.78 (1.63, 4.77)	
Severe (< − 5)	4	1.74 (0.18, 16.9)		3.10 (0.27, 35.9)	
FEV_1_ (*Z*)					
Normal (≥ − 1.64)	590	Reference	0.002	Reference	0.03
Mild (< − 1.64 to ≥ − 3)	364	1.62 (1.17, 2.24)		1.36 (0.95, 1.94)	
Moderate (< − 3 to ≥ − 5)	200	1.95 (1.34, 2.85)		1.61 (1.06, 2.45)	
Severe (< − 5)	11	1.93 (0.50, 7.40)		4.68 (1.12, 19.6)	
FVC (*Z*)					
Normal (≥ − 1.64)	708	Reference	< 0.001	Reference	< 0.001
Mild (< − 1.64 to ≥ − 3)	341	1.85 (1.35, 2.53)		1.77 (1.26, 2.48)	
Moderate (< − 3 to ≥ − 5)	111	2.37 (1.52, 3.71)		2.59 (1.59, 4.21)	
Severe (< − 5)	5	1.29 (0.14, 11.6)		2.61 (0.27, 25.5)	
FEV_1_/FVC (*Z*)					
Normal (≥ − 1.64)	802	Reference	0.19	Reference	0.52
Mild (< − 1.64 to ≥ − 3)	199	1.09 (0.74, 1.60)		0.81 (0.53, 1.23)	
Moderate (< − 3 to ≥ − 5)	151	1.47 (0.98, 2.19)		0.91 (0.58, 1.44)	
Severe (< − 5)	13	0.34 (0.04, 2.63)		0.28 (0.03, 2.27)	

*D*_LCO_, diffusing capacity of the lung for carbon monoxide; *K*_CO_, transfer coefficient of the lung for carbon monoxide; *V*_A_, alveolar volume; FEV_1_, forced expiratory volume in one second; FVC, forced vital capacity

^a^Adjusted for age, sex, smoking status, body mass index, First Nations status, and respiratory disease

When we limited the gas diffusing capacity data to those whose FEV_1_ and FVC values were within the clinically normal range (*n* = 544 patients including 26 deceased), *D*_LCO_ (*p* = 0.01) and *V*_A_ (*p* = 0.04) were still significantly associated with cardiovascular disease, while *K*_CO_ (*p* = 0.14) was not. Additional analysis showed no effect modification of abnormal FEV_1_/FVC (< 0.7) on gas diffusing capacity values and associations with cardiovascular disease.

### Mortality and Survival Distributions

**Fig. 2:**
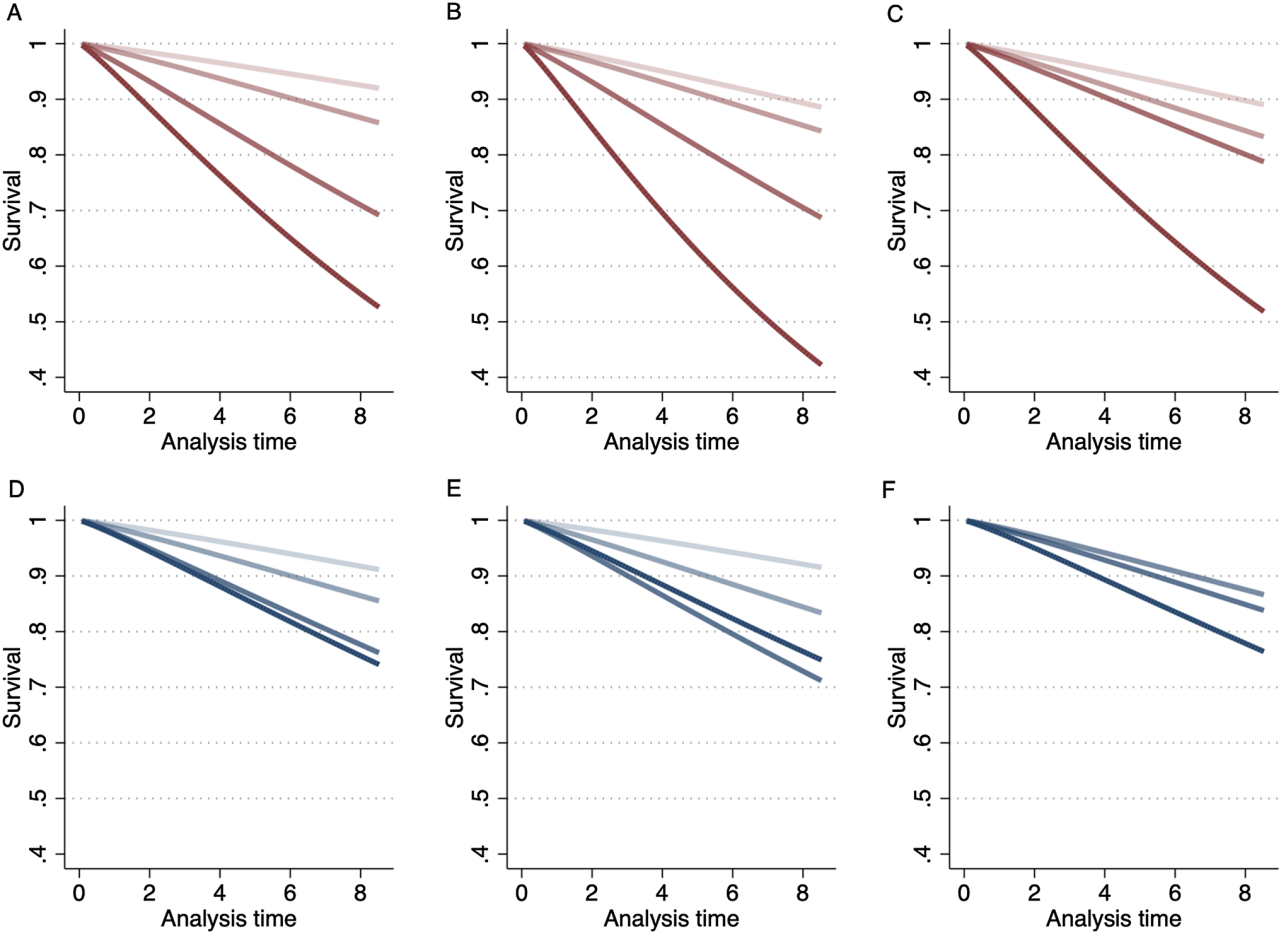
Survival estimates for **A** *D*_LCO_, **B** *K*_CO_, **C** *V*_A_, **D** FEV_1_, **E** FVC, and **F** FEV_1_/FVC Z-score tiers [Severity tiers for *Z*-scores; normal (> − 1.64), mild (< − 1.64 to − 3), moderate (− 3 to − 5) and severe (< − 5) are shown on graph as lightest to darkest] over time.* D*_LCO_ diffusing capacity of the lung for carbon monoxide, *K*_CO_ transfer coefficient of the lung for carbon monoxide, *V*_A_ alveolar volume, *FEV*_1_ forced expiratory volume in one second, *FVC* forced vital capacity

By August 2020, 108 (9%) patients had died. Respiratory-related death occurred in 42 (39%) patients, cardiovascular-related death in 25 (23%) patients, and other causes of death in 15 (14%), while we were unable to retrospectively determine cause of death in 16 (15%) patients. Adjusted survivor functions for gas-diffusing capacity and spirometry *Z*-score tiers are graphed in Fig. 2. The set of four curves appeared similar between gas diffusing capacity and spirometry tests, although the proportion of patients surviving appeared significantly less for severe *D*_LCO_, *K*_CO_, or *V*_A_ grading. Of 84 patients with a *D*_LCO_ below the lower limit of normal at baseline, only two had a lower *K*_CO_ *Z*-score, and ten a lower *V*_A_ *Z*-score.

### Multivariable Survival Modelling

*D*_LCO_ (HR = 2.99, 95% CI 1.83, 4.90), *K*_CO_ (HR = 2.14, 95% CI 1.38, 3.32), *V*_A_ (HR = 1.75, 95% CI 1.18, 2.58) *Z*-scores below the lower limit of normal were associated with increased risk of all-cause mortality after adjustment for age, sex, smoking status, body mass index, First Nations status, and respiratory disease. Full per-tier modelling is available in Table 4, and adjusted survivor functions are plotted in Fig. 2.

**Table 4:** Unadjusted and adjusted HRs with 95% confidence intervals for mortality from Cox regression for baseline lung function *Z*-scores

		Unadjusted		Adjusted^a^	
	*n*	HR (95% CI)	*p*	HR (95% CI)	*p*
*D*_LCO_ (*Z*)					
Normal (≥ − 1.64)	697	Reference	< 0.001	Reference	< 0.001
Mild (− 1.64 to − 3)	270	2.47 (1.40, 4.35)		1.86 (1.03, 3.34)	
Moderate (− 3 to − 5)	147	7.49 (4.48, 12.5)		4.40 (2.51, 7.69)	
Severe (< − 5)	51	15.0 (8.45, 26.5)		7.86 (3.84, 16.1)	
*K*_CO_ (*Z*)					
Normal (≥ − 1.64)	901	Reference	< 0.001	Reference	< 0.001
Mild (< − 1.64 to ≥ − 3)	149	2.49 (1.47, 4.24)		1.39 (0.79, 2.44)	
Moderate (< − 3 to ≥ − 5)	97	6.32 (3.99, 10.0)		3.11 (1.82, 5.32)	
Severe (< − 5)	18	14.1 (7.31, 27.1)		6.97 (3.08, 15.8)	
*V*_A_ (*Z*)					
Normal (≥ − 1.64)	753	Reference	0.011	Reference	0.014
Mild (< − 1.64 to ≥ − 3)	324	1.49 (0.98, 2.26)		1.59 (1.03, 2.45)	
Moderate (< − 3 to ≥ − 5)	84	2.40 (1.39, 4.16)		2.09 (1.19, 3.66)	
Severe (< − 5)	4	2.81 (0.39, 20.4)		5.75 (0.76, 43.6)	
FEV_1_ (*Z*)					
Normal (≥ − 1.64)	590	Reference	< 0.001	Reference	< 0.001
Mild (< − 1.64 to ≥ − 3)	364	1.83 (1.05, 2.84)		1.68 (1.00, 2.83)	
Moderate (< − 3 to ≥ − 5)	200	4.07 (2.55, 6.50)		2.98 (1.79, 4.96)	
Severe (< − 5)	11	2.90 (0.69, 12.2)		3.46 (0.78, 15.4)	
FVC (*Z*)					
Normal (≥ − 1.64)	708	Reference	< 0.001	Reference	< 0.001
Mild (< − 1.64 to ≥ − 3)	341	2.04 (1.32, 3.16)		2.10 (1.33, 3.30)	
Moderate (< − 3 to ≥ − 5)	111	3.68 (2.24, 6.05)		4.00 (2.37, 6.76)	
Severe (< − 5)	5	2.34 (0.32, 17.1)		3.40 (0.44, 26.3)	
FEV_1_/FVC (Z)					
Normal (≥ − 1.64)	802	Reference	< 0.001	Reference	0.63
Mild (< − 1.64 to ≥ − 3)	199	1.33 (0.80, 2.24)		1.00 (0.58, 1.69)	
Moderate (< − 3 to ≥ − 5)	151	2.55 (1.61, 4.04)		1.22 (0.74, 2.02)	
Severe (< − 5)	13	4.55 (1.42, 14.6)		2.01 (0.60, 6.79)	

*Z*-scores are derived from Global Lung Function Initiative reference values [20]

*D*_LCO_,  diffusing capacity of the lung for carbon monoxide; *K*_CO_, transfer coefficient of the lung for carbon monoxide; *V*_A_ alveolar volume; FEV_1_, forced expiratory volume in one second; FVC, forced vital capacity

^a^Adjusted for age, sex, smoking status, body mass index, First Nations status, and respiratory disease

Analysis restricted to only patients with normal FEV_1_ and FVC values showed that *D*_LCO_ (*p* = 0.03) was still associated with increased overall mortality risk, while *K*_CO_ (*p* = 0.16) and *V*_A_ (*p* = 0.08) were not. Further analysis showed no effect modification of abnormal FEV_1_/FVC (< 0.7) on gas diffusing capacity values and survival.

### Comparison to Spirometry

Median spirometry values were above the lower limit of normal (− 1.64 *Z*-score), with 575 (49%) FEV_1_, 457 (39%) FVC, and 363 (31%) FEV1/FVC results falling below the lower limit. Abnormal FEV_1_ (OR = 1.45, 95% CI 1.06, 1.97) and FVC (OR = 1.93, 95% CI 1.42, 2.63) were both associated with cardiovascular disease, with adjusted effect sizes for each tier similar to those seen for gas diffusing capacity measurements (Table 3). Abnormal FEV_1_ (HR = 2.21, 95% CI 1.41, 3.47), and FVC (HR = 2.57, 95% CI 1.71, 3.86) were also associated with elevated mortality risk, with adjusted effect sizes similar or less than equivalent gas diffusing capacity tiers (Table 4). We observed no associations between FEV_1_/FVC and either cardiovascular disease prevalence (OR = 0.84, 95% CI 0.60, 1.18) or elevated mortality risk (HR = 1.13, 95% CI 0.75, 1.70). In the subgroup of patients with normal spirometry, data relating to their gas diffusing capacity values with cardiovascular morbidity and adjusted survival curves and modelling were presented above.

## Discussion

In this study of 1165 predominantly First Nations Australian patients seen at specialist respiratory outreach clinics in regional and remote Queensland, we found that *D*_LCO_, *K*_CO_, *V*_A_, FEV_1_, and FVC values below the clinically normal range were associated with cardiovascular disease and mortality. This finding was independent of factors such as age, sex, personal smoking, BMI, and concurrent respiratory disease. Mortality risk almost doubled for each category increase in *D*_LCO_, *K*_CO_, and *V*_A_ tier (normal, mild, moderate, and severe) based on *Z*-scores in unadjusted but not adjusted models. While two other studies [6, 7] have reported similar findings with respect to the association of low diffusing capacity with future mortality, our study provides the first evidence that the *K*_CO_ and *V*_A,_ in addition to the *D*_LCO_, are each independent prognostic markers of mortality. Further, our data suggests estimated survival is similar or lower for gas diffusing capacity compared to equivalent spirometry *Z*-score tiers, suggesting that the former is more sensitive than the latter.

Neas et al. investigated pulmonary function as a predictor of mortality using First National Health and Nutrition Examination Survey (NHANES I) data [6]. Their analysis showed each 10% decrement in *D*_LCO_ percentage predicted below the lower limit of normal was associated with increased mortality [risk ratio (RR) = 1.33, 95% CI 1.21, 1.45], with a greater effect size than for either FEV_1_ (RR = 1.15, 95% CI 1.10, 1.19) or FVC (RR = 1.15, 95% CI 1.10, 1.20) [6]. This association persisted after including only participants with an FEV_1_ > 90% predicted (RR = 1.30, 95% CI 1.08, 1.56) [6]. Our similar analysis with stricter criteria, requiring both normal FEV_1_ and FVC, support and extend this existing evidence and showed *D*_LCO_ values below the lower limit of normal were still significantly associated with mortality. Our data suggest these associations with both all-cause mortality and cardiovascular disease occur independently of FEV_1_/FVC.

Recent analysis by Miller and Cooper [7] explored several grading schemes and showed the ATS/ERS four-tier system in combination with GLI reference equations [9] was best related to survival in a large (*n* = 13,829) clinical population and so were chosen for our analysis. Miller and Cooper reported a similarly large increase in risk related to *D*_LCO_ *Z*-score abnormality (mild: HR = 2.0, 95% CI 1.8, 2.1; moderate: HR = 3.4, 95% CI 3.2, 3.7; severe: HR = 6.2, 95% CI 6.0, 7.3) [7] and these hazard ratios were consistently higher than those for FEV_1_ and FVC across each tier of *Z*-score grading [7]. In concordance in our study, the largest effect across both cardiovascular morbidity and survival analysis were seen in the *D*_LCO_, except for the mild tier in which there was significant variation. Our effect size estimates have wide confidence intervals that make differences in the associations of outcomes with gas diffusing capacity and spirometry measurements difficult to discern, however estimates for the severe *Z*-score tier appear much larger for gas diffusing capacity measurements than for spirometry. Taken together with previously published evidence [6, 7], our study supports that associations between cardiovascular morbidity and overall survival to *D*_LCO_, *K*_CO_, *V*_A_ are similar or stronger than for equivalent FEV_1_, FVC, and FEV_1_/FVC values.

Our data supports a small but growing body of evidence that suggests that lung function impairment, as measured by spirometry and gas diffusing capacity, is associated with poor patient outcomes and this remains true in the absence of diagnosed respiratory disease [4, 6]. The limitations of diagnostic labels have, in part, given rise to the treatable traits paradigm, where clinical interventions are guided by genetic, phenotypic, and biomarker characteristics amongst others [22, 23]. Airflow limitation, eosinophilic airway inflammation, and emphysema [22] are examples of treatable traits that are identifiable using lung function testing, and our findings suggest these tests could also be applied outside of the respiratory clinic domain. Portability, (relative) low cost, standardisation, and well-defined guidelines for interpretation are key strengths of lung function tests that enable their use in a range of settings, including within office-based and outreach clinics. Broad application of the treatable traits approach necessitates the use of identification markers that are not isolated to large tertiary facilities in urban centres, but those that are ubiquitous and practical across the spectrum of clinical settings.

Our study has several important limitations. Firstly, we cannot discount the possibility of spectrum bias influencing our data, owing to the large number of clinic locations throughout Queensland. We are also reliant on mortality data reported in paper charts and electronic medical records, and so there may be a minority of deaths in this cohort that have not been accounted for. Secondly, although the GLI-2012 ‘other/mixed’ reference category has been found to be suitable for use in First Nations Australian children and young adults, we cannot be certain about its suitability to the adult population. GLI-2017 reference values were developed using participants of European ancestry and no ethnic correction is available [20]. It is unlikely these values are suitable for use in First Nations Australians. Thirdly, although we found significant associations, our data is limited by sample size, particularly within the severe *Z*-score tier of some measurements. This limits our ability to compare associations with outcome between gas diffusing capacity and spirometry measurements. Finally, our data includes adults seen in regional and remote Queensland, and so we cannot be certain about the generalisability of our findings to other populations more broadly.

In conclusion, our data suggests *D*_LCO_, *K*_CO_, and *V*_A_ measurements below the clinically normal range are associated with cardiovascular disease and all-cause mortality. These associations are independent of factors such as age, personal smoking, and concurrent respiratory disease and persist even when spirometry values are normal. Our data also shows *D*_LCO_, *K*_CO_, and *V*_A_ measurements were associated with similar or elevated mortality risk compared to spirometry equivalent spirometry *Z*-score tiers. Overall, *D*_LCO_, *K*_CO_, and *V*_A_ are prognostic markers of cardiovascular disease and all-cause mortality, with *D*_LCO_ the most capable measurement for this purpose as an additional prognostic marker available to clinicians.
